# Mapping of courses on vector biology and vector-borne diseases systems: time for a worldwide effort

**DOI:** 10.1590/0074-02760160295

**Published:** 2016-10-13

**Authors:** Jérôme Casas, Claudio Lazzari, Teresita Insausti, Pascal Launois, Florence Fouque

**Affiliations:** 1 Université de Tours, Institut de Recherche en Biologie de l’Insectes, Unité Mixte de Recherches, Centre National de la Recherche Scientifique, Tours, France; 2 Research and Training in Tropical Diseases, World Health Organization, Geneva, Switzerland

**Keywords:** teaching, expertise, entomology, MOOCs, vector control, insect control, crisis management

## Abstract

Major emergency efforts are being mounted for each vector-borne disease epidemiological crisis anew, while knowledge about the biology of arthropods vectors is dwindling slowly but continuously, as is the number of field entomologists. The discrepancy between the rates of production of knowledge and its use and need for solving crises is widening, in particular due to the highly differing time spans of the two concurrent processes. A worldwide web based search using multiple key words and search engines of onsite and online courses in English, Spanish, Portuguese, French, Italian and German concerned with the biology of vectors identified over 140 courses. They are geographically and thematically scattered, the vast majority of them are on-site, with very few courses using the latest massive open online course (MOOC) powerfulness. Over two third of them is given in English and Western Africa is particularity poorly represented. The taxonomic groups covered are highly unbalanced towards mosquitoes. A worldwide unique portal to guide students of all grades and levels of expertise, in particular those in remote locations, is badly needed. This is the objective a new activity supported by the Special Programme for Research and Training in Tropical Diseases (TDR).

While infection with the Chikungunya virus has not reached neither its southward nor northward limits yet, the Zika virus, transmitted mainly by the same mosquito, *Aedes aegypti*, is already creating another unprecedented outbreak in the Americas. Zika was declared a Public Health Emergency of International Concern (PHEIC) by WHO in February 2016 (Gulland 2016), due to the consequences on newborn babies with microcephaly (da Silva & Gao 2016). Another mosquito borne epidemic is also in the making in Africa, with the yellow fever outbreak in Angola causing hundreds of deaths (Butler 2016). While major emergency efforts are being mounted for each crisis anew (WHO 2016), it becomes evident that we should swiftly move from case by case responses to a sustainable response endeavor. Systemic sustainability can only function if there is a knowledge capital to tap into. Unfortunately, knowledge about the biology of the vectors is dwindling slowly but continuously, weakening therefore our response capacity. This situation is also reflected in the decline in the number of professional entomologists worldwide and in the change of their fields of expertise, further remote from biology control into genomics for example (Reisen 2014). These new fields have shifted attention and funding away from areas which could provide more immediate and oftentimes less expensive solutions (Lambrechts et al. 2009, Mullens et al. 2015). This impacts directly the teaching activities the scientists are responsible for. England, for example, has no master degree in entomology anymore and many of its most famous institutions have been closed (in particular the Imperial College Department of Entomology, Leather 2009, Loxdale 2016). France had over 50% of the workforce over 50 years old in 2004 (Cuisance & Rioux 2004), now well into retirement. In the USA, the number of doctorate students in insect systematics was predicted in 1995 to decline to null in 2017 (Daly 1995). Finally, India needs to educate over one thousand vector biologists to cover its 643 districts, stating from some 100 as of today (Pandey et al. 2015). The Zika epidemy revealed that WHO has only a handful entomologists in all its branches left (personal counting). The number of viruses already proved to be hosted by insects in the wild and presenting possible health problems for humans and animals and eventually breaking out their localised regions due to human progression in remote locations is by contrast large (see for example the nearly 40 mosquito-borne flaviviruses listed in Weissenböck et al. 2010). It is thus time to assess our remaining research and educational potential for mounting systemic worldwide responses against vector born diseases. This paper is a worldwide stock taking focusing on on-site and on-line courses on vector biology and vector-borne diseases.

The objective of this mapping exercise was to identify training courses on either distance learning (online) and on-campus courses in different languages, i.e., English, Spanish, French, Portuguese, German and Italian by using two distinct search methodologies on the web. The first one was in analysing the results returned by different search engines, without preconceived hierarchy between them (Google, Yahoo, Bing) using the following main key words and their variations: Vector borne diseases; Arthropod vectors; Vector Biology; Vector Ecology; Insect vectors; Disease vectors; Medical Entomology; Applied Entomology; Molecular Entomology; Parasitology - Parasitic diseases; Trypanosomiasis; Dengue; Malaria; Chikungunya; Schistosomiasis; Bugs; Mosquito; Tsetse; Tick.

We did not use names of parasites, as our study focused on vectors and not the pathogens they transmit, nor generic words such as ‘vector’, because generic names delivered unmanageable amount of information coming from different disciplines. Diseases other than those listed (for example, Rift valley virus, Zika) do have vectors which are found using other combinations of key words. The second entry was in visiting the websites of universities and research organisms, country by country and looking directly into their pages for those which offer courses and degrees in the area of biology. There might exist courses on site with no information available online, but we believe this to be a minority of cases. While we strove for a systematic coverage and checked carefully hundreds of universities, there is indeed no certainty we have covered all possible places. By contrast, we are certain to have covered all major universities and the vast majority of medium-sized universities. We understand the use of the word “courses” as being teaching blocks leading to a diploma at any level of education; thus, our search included master degrees, postgraduate courses, courses given to technicians etc. Not a single university for which the above key words were found was left unchecked. The search was done for each language, with the key words duly translated to the pertinent language. While English is covering the largest portion, in particular for higher education, French was picked up to cover the francophone Africa as well as some countries of Asia, such as Vietnam and Cambodgia, Spanish for covering Central and South America, and Portuguese for covering lusophone Africa and Brazil. We added German and Italian due to our command of these two languages. The survey, while quite extensive, thus does not cover the entire world. All synonyms were employed (not listed here). Some universities have master degrees in entomology, but they do not include vectors in the program. These were not included in the analysis. For instance, courses on general entomology, parasitology or tropical medicine that did not mention vectors or equivalents at all were not included. If any key word related to our topic appeared in the program, the program was included in the analysis, also in the case their treatment was only marginal. Courses dedicated to the sole medical or pest control topics were excluded of the analysis. We did not search in Russia and its neighbor countries, nor in Japan. China was not covered either, but we located one international course. No automatic search algorithm was used. The dynamic and iterative way of conducting the survey, the joint use of several key words to reduce the immense number of results, the decrease of relevance of the ordered list of results found in each search and the inclusion of positive results rather than the exclusion of negative ones imply that the procedure cannot be summed up by a flow chart or decision tree. The main survey was carried out by a single experienced researcher. Then, in order to ensure full coverage, the results were thoroughly checked by a second experienced researcher and the outcome was eventually checked again, through random sampling, by the other authors. The work was carried out over a period of some four months, starting in September 2015. The total number of hours spent is about 600 h.

Some 140 courses of interest were identified, over 70% of them being organised by universities. Joint courses between countries are very rare, as courses given in two languages. The geographic spread of institutions shows a worldwide interest in global issues related to vector biology and vector diseases. Western Africa is particularly poorly represented, while South America evenly covered. The Mediterranean border, while poorly represented, has one course spanning many countries.

The continental distribution of courses shows (Fig. 1) a bias towards South and Central America and North America. Asia is nearly on par with Europe. In terms of language, English makes up more than two thirds of the worldwide offer, followed by Spanish. The remaining languages are minor ones. The vast majority (nearly 90%) has only on-site courses (Fig. 2, black), a bit more than half a dozen is offered online (light gray) and only two are mixed (dark grey). Online courses remain thus a rarity and few seem to be riding the latest massive open online courses (MOOCs) wave.

**Fig. 1:**
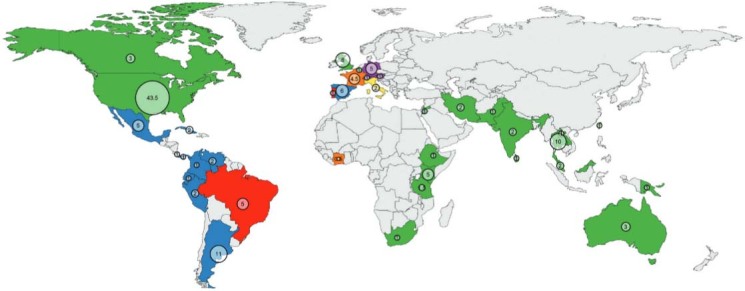
: courses offered by country and language. The colour represents the language of the course (English in green, Portuguese in red, Spanish in blue, French in orange, Italian in yellow and German in violet) and the circle is proportional to the number of courses.

**Fig. 2:**
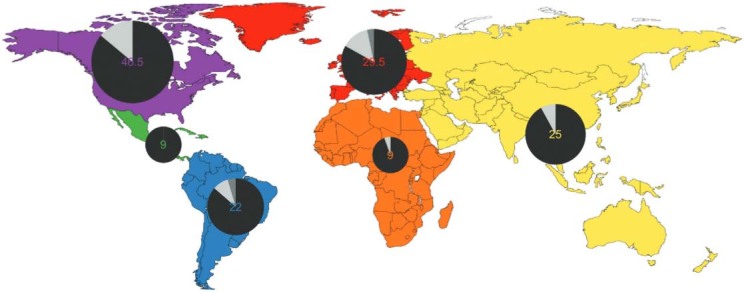
: courses offered by continents. The colour represents the continent and the circle is proportional to the number of courses. Onsite courses are in black, mixed in dark grey and online in light grey.

The results of the mapping deliver three lessons. First, it is quite difficult to find a training course tailored to the specific needs of the prospective student and nearly impossible to evaluate the contents in detail. The breadth and depth of the offered courses worldwide is indeed strikingly heterogeneous. Searching for specific groups of organisms, with mosquitoes as sole exception, is a vain endeavor despite being a most natural way of searching. The widely varying and oftentimes rudimentary presentation of the courses did not enable us to make a comprehensive mapping of the number of hours devoted to specific topics, control methods or groups of organisms, except that mosquitoes are by large the most commonly treated group. Prices vary by several orders of magnitude, without a clear relationship with teaching quality or depth. Second, the mismatch between teaching offer and educational demand to tackle urgent problems implies that a survey of existing forces and a plan for capacity building is needed, using previous lessons from past and current epidemics. Indeed, we observed that many scientists and organisations carrying scientific work in this area and publishing in the best journal do not offer courses based on their expertise. In the face of declining number of knowledgeable scientists and vast demand, this capital cannot remain untapped. Among the possible reasons for this state of affairs, we identified their topic-wise isolation of these scientists within their institutions. The critical mass needed to offer substantial teaching material and/or manage a comprehensive web site was not reached then. Finally, a global effort in education will have to resort to a combination of face to face teaching and innovative MOOCs. The scope for progress on this front is huge.

We therefore conclude that the field of vector biology, at the world scale, is in an urgent need of harmonisation, consolidation and modernisation, with state of the art teaching material and courses design dedicated towards specific groups of organisms and specific epidemiological questions. A worldwide unique portal to guide students of many different backgrounds and expectations, in particular those in remote locations, would be a most welcome crystallisation nucleus of such efforts.
